# Direct Conjugation of Streptavidin to Encoded Hydrogel Microparticles for Multiplex Biomolecule Detection with Rapid Probe-Set Modification

**DOI:** 10.3390/polym12030546

**Published:** 2020-03-03

**Authors:** Yoon Ho Roh, Ju Yeon Kim, Seok Joon Mun, Hye Sun Lee, Changhyun Hwang, Kyong Hwa Park, Ki Wan Bong

**Affiliations:** 1 Department of Chemical and Biological Engineering, Korea University, Seoul 02841, Korea; yoonho90@korea.ac.kr (Y.H.R.); 7777kmg@hanmail.net (J.Y.K.); msj95@naver.com (S.J.M.); changhwang77@gmail.com (C.H.); 2 Division of Oncology/Hematology, Department of Internal Medicine, Korea University College of Medicine, Seoul 02841, Korea; hyesunxox@naver.com (H.S.L.); khpark@korea.ac.kr (K.H.P.)

**Keywords:** encoded hydrogel microparticle, streptavidin, aza-Michael addition click reaction, stop flow lithography, probe-set modification

## Abstract

Encoded hydrogel microparticles synthesized via flow lithography have drawn attention for multiplex biomarker detection due to their high multiplex capability and solution-like hybridization kinetics. However, the current methods for preparing particles cannot achieve a flexible, rapid probe-set modification, which is necessary for the production of various combinations of target panels in clinical diagnosis. In order to accomplish the unmet needs, streptavidin was incorporated into the encoded hydrogel microparticles to take advantage of the rapid streptavidin–biotin interactions that can be used in probe-set modification. However, the existing methods suffer from low efficiency of streptavidin conjugation, cause undesirable deformation of particles, and impair the assay capability. Here, we present a simple and powerful method to conjugate streptavidin to the encoded hydrogel microparticles for better assay performance and rapid probe-set modification. Streptavidin was directly conjugated to the encoded hydrogel microparticles using the aza-Michael addition click reaction, which can proceed in mild, aqueous condition without catalysts. A highly flexible and sensitive assay was developed to quantify DNA and proteins using streptavidin-conjugated encoded hydrogel microparticles. We also validated the potential applications of our particles conducting multiplex detection of cancer-related miRNAs.

## 1. Introduction

Encoded particle (bead)-based assays have attracted considerable attention for biomolecule detection due to their fluid-phase kinetics with respect to target–probe interaction, which has better efficiency than the solid-phase kinetics of planar arrays [1]. Silica [2,3] and polystyrene [4,5] particles are conjugated with capture probes on the surface and are encoded spectrally, physically, or graphically for multiplex detection of biomolecules [6]. As a representative encoded particle-based assay, Luminex technology with polystyrene microspheres spectrally encoded with a combination of red and near-infrared fluorophores has been widely used for multiplex bioassays [7]. However, the polymer surfaces on which the probes are immobilized are not ideal for biological interactions because two-dimensional rigid polymer surfaces cause steric hindrance and limited loading density of probes [8]. In addition, probes exposed on surfaces may lose their native structure and function, which impairs the sensitivity, specificity, and dynamic range of biomolecule detection [9]. 

Hydrogel-based microparticles have several advantages over silica or polystyrene microparticles as hydrogel consists of three-dimensional mesh-like scaffolds [10]. Their semi-solid structure provides increased probe-loading density and enhanced binding kinetics without consideration of steric hindrance, resulting in a 10-fold higher sensitivity than that of solid surfaces [11]. In addition, hydrogel microparticles have been spectrally or graphically encoded for their use in multiplex bioassays [12,13,14]. Among the various encoded hydrogel microparticles, the barcoded hydrogel microparticles have gained considerable attention due to their high multiplexing capacity of up to 10^5^ attributed to the engraving of complex graphical codes in a single particle [14]. Generation of graphically-encoded hydrogel microparticles was enabled via the stop flow lithography (SFL) technique, in which a short burst of photomask-defined UV is selectively exposed to a monomer stream for the fabrication of particles with pre-defined shapes [15]. For the incorporation of probes onto the barcoded hydrogel microparticles, acrylate-modified probes were mixed with monomers in order to be immobilized in hydrogel networks during the particle synthesis step [16]. However, immiscibility between probes and hydrophobic photoinitiators in the monomer causes aggregation of the probes [17], and the low copolymerization yield of SFL allows only ~10% utilization of the input probes [8]. To overcome these problems arising from probe incorporation during particle synthesis, a post-synthesis functionalization process that conjugates probes using unreacted double bonds (C=C) remaining inside the hydrogel particles was developed [18]. However, for the practical application of encoded hydrogel microparticles in clinical trials challenges persist as it is difficult to achieve a rapid probe-set modification—with the present probe incorporation methods—which is highly desired for the preparation of various combinations of target panels to obtain more accurate diagnostic results [19,20]. This is because once the probe is attached to the particles, the entire process, from the particle synthesis to the probe conjugation, must be repeated in order to modify the probe-set.

The streptavidin–biotin system is one of the notable approaches to overcome the issues associated with flexible probe-set modifications as the streptavidin–biotin complex has high binding rate constants (~10^7^ M^−1^ s^−1^) [21] that enables a rapid probe-set modification (e.g., interaction between biotinylated probes and streptavidin-coated substrates). The streptavidin–biotin system is also suitable for use in various biomedical applications due to the specific interactions which remain stable even in potentially adverse environments due to the presence of denaturing enzymes, unfavorable pH and temperatures, and other denaturing reagents [22,23,24]. Consequently, substantial efforts have been made to adapt the streptavidin–biotin system to encoded hydrogel microparticles to achieve a highly flexible probe-set modification in addition to the high assay performance. For this purpose, acrylate-modified streptavidin was incorporated into the monomer and conjugated during the particle synthesis [25]. However, due to the immiscibility of streptavidin and the hydrophobic photoinitiator in the monomer, the loading density of streptavidin is limited up to 0.4 mg/mL which is insufficient for further target quantification. Unlike previous method, encoded hydrogel microparticles including carboxyl groups enabled streptavidin conjugation with a high loading density. However, the charged carboxyl groups cause undesired swelling and shape deformation of hydrogels [26]. In addition, charged species can affect the target accessibility to probes, which impairs the assay sensitivity and specificity [27]. Furthermore, the 1-ethyl-3-(3-(dimethylamino) propyl) carbodiimide (EDC)/N-hydroxysuccinimide (NHS) reaction, which is used for all the above-mentioned methods of streptavidin conjugation, has low efficiency due to the instability of the NHS ester in aqueous conditions with a short hydrolysis half-life of less than an hour [28].

In this study, we presented a novel method of streptavidin conjugation to the encoded hydrogel microparticles to fully exploit the high assay capability and achieve a rapid probe-set modification. This goal was accomplished through the aza-Michael addition click reaction that involves the formation of bonds between primary amine groups of streptavidin and unreacted double bonds in the encoded hydrogel microparticles under a mild reaction condition. We showed that the streptavidin was well incorporated into the encoded hydrogel microparticles and demonstrated the formation of bonds using spectroscopy. We demonstrated the high assay capability and flexibility of streptavidin-conjugated encoded hydrogel microparticles from the proof-of-concept studies, by quantifying target DNA and protein. We also demonstrated the potential applications of our particles by performing multiplex detection of cancer-related miRNAs.

## 2. Experimental Section

### 2.1. Fabrication of the Polydimethylsiloxane (PDMS) Microfluidic Device

A microfluidic device was first designed using AutoCAD (Autodesk, San Rafael, CA, USA) and printed on photomask films (Han and All Technology, Ansan-si, Gyeonggi-do, Korea). The SU-8 master mold was generated through photolithography using silicon wafers coated with SU-8 25 (Microchem, Westborough, MA, USA), which serve as a negative photoresist. The SU-8 25 thickness was adjusted to 24 μm. PDMS (Sylgard 184, Corning, Midland, MI, USA) mixed with the curing agent at a *w*/*w* 10:1 ratio was poured on a SU-8 master mold and cured at 70 °C for 8 h. Then, the PDMS slab was detached from the master mold and punched with a 1 mm or a 10 mm biopsy punch (Miltex, Princeton, NJ, USA) to create holes for inlet and outlet reservoirs, respectively. After preparing the PDMS slab, a glass slide was coated with PDMS and partially cured at 70 °C for 1 h. The PDMS slab was attached to the glass slide and baked overnight for complete curing.

### 2.2. Stop Flow Lithography (SFL) Setup

A UV pressure synchronization system controlled by a homemade circuit board and a LabView (National Instruments, Austin, TX, USA) script were used to perform SFL for hydrogel microparticle fabrication. The precursor solution was loaded into the microfluidic device, which was mounted on an inverted microscope (Axiovert 200, Zeiss, Oberkochen, Germany), and injected into the device under controlled air pressure using a pressure regulator (ITV0031-3BL, SMC, Tokyo, Japan). A photomask with various patterns (25,000 dpi) was inserted into the field-stop of the microscope. As a photopolymerization source, a LED lamp (Mounted High-Power LEDs M365L2, Thorlabs, Newton, NJ, USA) was used and controlled by a LED driver (T-cube High Power LED driver, Thorlabs). UV intensity was kept constant at 2200 mW/cm^2^.

### 2.3. Fabrication of Streptavidin-Conjugated Encoded Hydrogel Microparticles

The precursor solutions for the fabrication of hydrogel microparticles were composed of 20% (*v*/*v*) polyethylene glycol diacrylate 700 (PEGDA 700), 40% (*v*/*v*) polyethylene glycol 600 (PEG600), 5% (*v/v*) Darocur 1173 (all from Sigma Aldrich, St. Louis, MO, USA), and 35% (*v*/*v*) deionized (DI) water. Encoded hydrogel microparticles were synthesized via SFL after loading the precursor solutions into the microfluidic device. SFL was performed with a repeated cycle that consisted of flow (400 ms), stop (200 ms), expose (60 ms), and hold (140 ms) times. Graphical codes were assigned to the particles by changing the photomasks. Synthesized particles were collected at the outlet of the microfluidic device and rinsed five times in 1x PBST in order to remove the macromers remaining inside the hydrogel microparticles and in the buffer prior to conjugation. Then, 50 μL of 5 mg/mL streptavidin (Thermo Fisher Scientific, Waltham, MA, USA) were dissolved in 1x PBS and incubated with 50 μL of particles (~120 particles/μL) at 25 °C for 24 h in a thermal shaker (Hangzhou Allsheng Instruments Co. Ltd, Hangzhou, China). The pH of the mixture was adjusted to ~8.5 with 0.5 M NaOH before incubation.

### 2.4. Characterization of Streptavidin-Conjugated Encoded Hydrogel Microparticles

To remove unreacted double bonds that remained inside the encoded hydrogel microparticles, we used SH-PEG800 (Sigma Aldrich) to proceed with the thiol-ene reaction between unreacted double bonds and thiol groups of the SH-PEG800. Encoded hydrogel microparticles suspended in 1x PBST (~30 particles/μL; 100 μL) were mixed with 100 μL of SH-PEG800 (10 mg/mL) at 37 °C for 4 h in a thermal shaker. Then, particles were mixed with 1 μL of FITC–PEG–SH (1 mg/mL) at 37 °C for 4 h to verify whether any unreacted double bonds remained inside the hydrogel networks. Untreated particles were used as a control.

Particles without post-processing and particles pre-reacted with SH–PEG were mixed with streptavidin and reacted according to the procedure described in Section 2.3. Throughout the reaction, ~120 particles were collected at 0.5, 1, 2, 4, 8, 12, 24, 48, and 72 h and reacted with 2 μL of FITC–PEG–biotin (2 mg/mL) at 25 °C for 0.5 h. To determine the amount of streptavidin incorporated into the encoded hydrogel microparticles, streptavidin concentrations of 0.625, 1.25, 2.5, and 5 mg/mL were mixed with particles and reacted according to a previously reported procedure. Then, the particles reacted with 2 μL of FITC–PEG–biotin (2 mg/mL) or with 1 μL of FITC–PEG–SH (1 mg/mL) to assess the amount of incorporated streptavidin and the number of remaining unconverted double bonds inside the hydrogel network, respectively.

### 2.5. Spectroscopic Analysis

Synthesized encoded hydrogel microparticles were freeze-dried before spectroscopic analysis. Fourier-transform infrared (FT-IR) spectra were recorded on a Cary 630 FT-IR (Agilent, Santa Clara, CA, USA). An average of 32 scans with a 2 cm^–1^ resolution was taken to obtain spectra. Raman spectroscopy was performed on an inVia Raman microscope (Renishaw, Wotton-under-Edge, UK) using a 787 nm near-IR diode laser with an operating power of 300 mW.

### 2.6. DNA and Protein Detection

To perform singleplex DNA detection, streptavidin-conjugated encoded hydrogel microparticles reacted with two types of biotinylated DNA probes. We assigned different graphical codes to each particle to identify targets. Specifically, encoded hydrogel microparticles (20 μL) suspended in 1x PBST (~30 particles/μL) were mixed with 20 μL of the biotinylated DNA probe (50 μM) at 25 °C for 2 h in the thermal shaker. After three rinses in 1x TET (1x TE buffer and 0.05% (*v*/*v*) Tween-20, all from Sigma Aldrich), 5 μL of FAM (fluorescein amidite)-attached target DNA (Integrated DNA Technology, Coralville, IA, USA) was added to a total volume of 50 μL at 0.1, 1, 10, and 100 nM concentrations. Each assay contained 350 mM NaCl with ~60 particles and was conducted at 25 °C for 2 h in a thermal shaker. For multiplex detection, two types of particles were added to the assay mixture and two types of 50 nM FAM-attached target DNA were added only in the assays containing the targets. The assay was conducted under the same conditions as for the singleplex detection.

Protein detection proceeded in a similar way as DNA detection. First, streptavidin-conjugated encoded hydrogel microparticles reacted with the biotinylated antibody specific to ovalbumin. A volume of 20 μL of hydrogel microparticles suspended in 1x PBST (~30 particles/μL) was mixed with 10 μL of biotinylated antibody (1 mg/mL) at 25 °C for 2 h in the thermal shaker. After three rinses in 1x PBST, 10 μL of Alexa 488-labeled ovalbumin (Thermo Fisher Scientific) was added at concentrations of 250, 62.5, 15.6, 3.9, 0.96 ng/mL in a total assay volume of 50 μL. Each assay contained ~60 particles and was conducted at 25 °C for 2 h in a thermal shaker.

### 2.7. Detection of Cancer-Associated miRNAs 

To conduct singleplex miRNA detection, two types of biotinylated DNA probes reacted with streptavidin-conjugated encoded hydrogel microparticles. The biotinylated DNA probes consisted of two parts: sequences that are specific to the target miRNA and those that are specific to the fluorescently-labeled universal adapter. We assigned a code 1 to miR-451a and a code 2 to miR-6880-5p. Hydrogel microparticles (10 μL) suspended in 1x TET (~30 particles/μL) were mixed with 5 μL of each cancer-related miRNA (Integrated DNA Technology) at 0.08, 0.4, 2, 10, and 50 nM concentrations in a total assay volume of 50 μL. Each assay reaction contained 200 mM NaCl and was conducted at 55 °C for 2 h in a thermal shaker. After three rinses with 1x TET containing 50 mM NaCl, 245 μL of the ligation mixture was added. The ligation mixture comprised 1350 μL 1x TET, 150 μL of NEBuffer 2 (New England Biolabs, Ipswich, MA, USA), 250 nM ATP (New England Biolabs), 800 U/mL T4 DNA ligase (Fermentas, Waltham, MA, USA), and 40 nM fluorescently-labeled universal adapter (Integrated DNA Technology). Then, the mixture was hybridized at 37 °C for 45 min in a thermal shaker. For multiplex detection, two types of particles (~60 particles per target) and two types of cancer-related 10 nM miRNA were added to the assay mixtures containing targets. The assay was conducted using the same procedure of the singleplex detection.

### 2.8. Image Analysis

RGB fluorescence images of the hydrogel microparticles were obtained using a digital single-lens reflex (DSLR) camera (Eos 6D, Canon, Tokyo, Japan). Monochrome fluorescence images of the particles were acquired using a metal-oxide semiconductor (sCMOS) camera (Prime, Photometrics, Tucson, AZ, USA). Both cameras were connected to an inverted microscope and used a fluorescence light source (Illuminator HXP 120 V, Zeiss). To obtain the signal of DNA and protein detection, 11 mW/cm^2^ LED intensity with 200 ms exposure time was used. The signal of miRNA detection was obtained with 11 mW/cm^2^ LED intensity with 400 ms exposure time. The monochrome fluorescence images were analyzed by Image J software (National Institutes of Health, Bethesda, MD, USA).

## 3. Results and Discussion

### 3.1. Synthesis and Characterization of Streptavidin-Conjugated Encoded Hydrogel Microparticles

**Figure 1:**
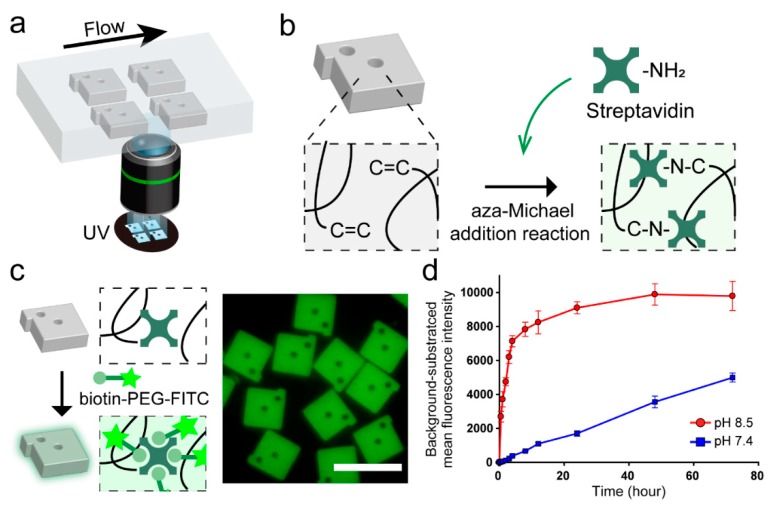
Schematic representation of the synthesis of streptavidin-conjugated encoded hydrogel microparticles and optimization of the streptavidin conjugation process. (**a**) Synthesis of hydrogel microparticles by stop flow lithography (SFL). (**b**) Conjugation of streptavidin using the aza-Michael addition reaction between the unreacted double bonds remaining in the hydrogel microparticles and primary amine groups in streptavidin. (**c**) Schematic view of streptavidin identification using biotin–PEG–FITC (left) and fluorescence micrograph of streptavidin-conjugated encoded hydrogel microparticles (right). Scale bar is 100 μm. (**d**) Optimization of streptavidin conjugation versus pH of the mixture and reaction time.

Encoded hydrogel microparticles were prepared using the SFL technique which can easily engrave different graphical patterns in the hydrogel microparticles by exposing a short burst of shape-defined UV to the monomer stream (Figure 1a). Each particle was graphically encoded with combinations of internal shape holes with a protruding bar. Encoding capacity could be easily expanded over 500 by changing the shape and the number of internal holes [18]. 

Due to the low monomer conversion of SFL, unreacted double bonds (C=C), which are mainly acrylate groups of PEGDA, remain inside the hydrogel networks after particle synthesis [29]. By using these double bonds, we previously demonstrated the functionalization of the hydrogel matrix with biological probes including thiolated DNA and antibodies via thiol-ene click reaction [17,18]. However, due to the absence of reactive groups of streptavidin (e.g., thiols) [30], we introduced the aza-Michael addition click reaction to directly conjugate streptavidin into the unreacted double bonds remaining in hydrogel microparticles. aza-Michael addition reaction is involved in the formation of a C–N bond between α,β-unsaturated compounds and nitrogen donors (Figure 1b) [31,32]. In our case, α,β-unsaturated compounds are unreacted double bonds, and nitrogen donors are primary amine groups of streptavidin. Although the aza-Michael addition reaction is not the part of the classically defined click reactions, their concepts are similar to that of click reactions including (1) proceeds in mild and aqueous conditions, (2) insensitivity to oxygen or water, and (3) applicability to biomolecules without producing hazardous substances, and thus has been considered to fit click criteria [32,33,34,35]. In addition, the reaction is one of the most widely used reactions for the addition of amines to acrylates, especially in protein modification [36,37]. To identify the presence of streptavidin inside the encoded hydrogel microparticles after the reaction, Biotin–PEG–FITC was used as a fluorescent marker (Figure 1c). We optimized the aza-Michael addition reaction by monitoring the kinetics of the streptavidin conjugation process as a function of the pH of the mixture and reaction time using biotin–PEG–FITC. At pH 8.5, the fluorescent intensity of the particles increased rapidly for 4 h and reached saturation in 48 h (Figure 1d). In contrast, at pH 7.4, the fluorescent intensity of the particles increased slowly with no saturation until the 72 h time point. This is because the isoelectric point of streptavidin is near neutral, and thus, the primary amine groups can act as nucleophiles above neutral pH [38]. It should be noted that all the reaction proceeded with a molar ratio of acrylate to amine at ~2:1 (Supplementary Materials).

With the optimized reaction condition, we verified the consumption of unreacted double bonds during the reaction by performing the aza-Michael addition reaction with streptavidin on two types of particles, particles without post-processing and particles that were pre-reacted with SH–PEG to block unreacted double bonds through the thiol-ene reaction (Figure S1, Supplementary Materials). The fluorescent intensity of unblocked particles showed the same response as the other ones (Figure 1d). Meanwhile, fluorescence intensity was not observed in pre-blocked particles within the monitored time range, meaning that the streptavidin is conjugated to the hydrogel particles through the reaction, with double bonds remaining inside the hydrogel networks. We further characterized the reaction for different streptavidin concentrations (Figure S2, Supplementary Materials). As expected, the fluorescent intensity due to biotin–PEG–FITC showed a linear response as a function of the increasing streptavidin concentration. In addition, FITC–PEG–SH was used to monitor the remaining unreacted double bonds, resulting in an opposite trend compared to that was observed in results from the biotin–PEG–FITC. Such a result is expected because the number of available unreacted double bonds proportionally decreases with the increase in streptavidin concentration. The loading density of streptavidin using the aza-Michael addition click reaction after the particle synthesis is 12.5-fold higher than that of the streptavidin-conjugated particles during particle synthesis [25]. In addition, we confirmed that the conjugated streptavidin remained stable for at least five weeks, indicating the suitability of adopting the aza-Michael addition click reaction to conjugate streptavidin in encoded hydrogel microparticles (Figure S3, Supplementary Materials).

### 3.2. Spectroscopic Analysis of Streptavidin-Conjugated Encoded Hydrogel Microparticles

**Figure 2:**
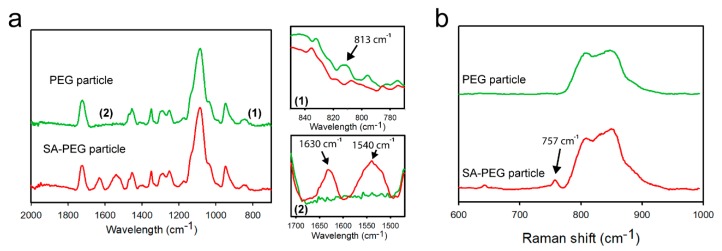
Spectroscopic analysis of encoded hydrogel microparticles: (**a**) FTIR spectra in the 700–2000 cm^−1^ region of encoded hydrogel microparticles before and after the reaction with streptavidin. (**b**) Raman spectra in the 600–1000 cm^−1^ region of encoded hydrogel microparticles before and after the reaction with streptavidin.

To clearly demonstrate the reaction between the unreacted double bonds and the primary amine groups, we characterized the formation or disappearance of bonds with FTIR-ATR and Raman spectroscopy. From the FTIR spectra of encoded hydrogel microparticles, we found that the distinct peak of the unreacted double bonds (C=C, 813 cm^−1^) remaining in the particles was clearly reduced after the reaction with streptavidin (Figure 2a) [39]. Meanwhile, two identifiable bands at 1630 cm^−1^ (amide I) and 1540 cm^−1^ (amide II) appeared in the streptavidin-conjugated encoded hydrogel microparticles, which confirms the presence of streptavidin in the particles after the reaction [40]. Moreover, we used Raman spectroscopy to verify the formation of C–N–C bonds because it provides strong intensity for the structure with symmetric stretches of bonds [41]. After the reaction, we found a distinct peak at 757 cm^−1^, which could be attributed to the vibration of C–N–C stretching (Figure 2b) [42]. These results confirm the formation of C–N–C bonds between the unreacted double bonds of the hydrogel microparticles and the primary amine groups of streptavidin. 

### 3.3. Assay Performance and Flexibility of the Streptavidin-Conjugated Encoded Hydrogel Microparticles

To assess the assay performance of streptavidin-conjugated encoded hydrogel microparticles, the probe density was first evaluated in particles as it is directly related to the sensitivity and dynamic range of the assay [43,44]. Biotinylated DNA probes were attached to hydrogel microparticles at various concentrations of incorporated streptavidin (Figure S2, Supplementary Materials). We conjugated a 6-FAM fluorescent dye at the 3′ end of DNA probes to quantitatively compare the number of probes. Fluorescence intensity increased linearly as a function of streptavidin concentration and achieved the highest signal at the maximum streptavidin concentration (5 mg/mL) representing the highest DNA probe concentration (Figure S4, Supplementary Materials). 

**Figure 3:**
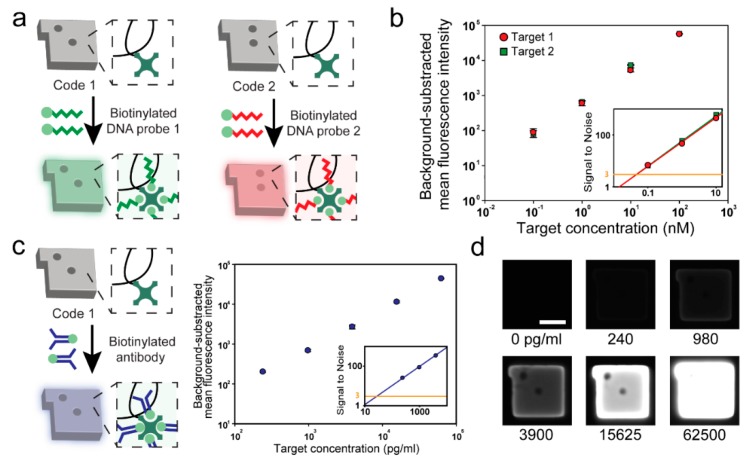
Assay performance and flexibility evaluation of streptavidin-conjugated encoded hydrogel microparticles. (**a**) Schematic view of the process used for the attachment of the biotinylated DNA probe to hydrogel microparticles. (**b**) Standard calibration curves of two DNA targets. (**c**) Schematic view of the process used for the attachment of biotinylated antibodies to hydrogel microparticles (left) and standard calibration curves for ovalbumin (right). (**d**) Monochrome micrograph of the particles at various ovalbumin concentrations. Scale bar represents 25 μm.

With the maximized probe density, we performed 2-plex DNA detection using two sets of streptavidin-conjugated encoded hydrogel microparticles. As these microparticles are ready-to-use without any deterioration in terms of the quality regardless of the storage time, the desired probe-set could be rapidly prepared through the streptavidin–biotin reaction from the stored particles (Figure 3a). The assay performance of the hydrogel microparticles was confirmed in singleplex detection before conducting the multiplex detection. We assessed the sensitivity of two types of FAM-labeled DNAs at concentrations ranging from 0.1 nM to 100 nM (Figure 3b). Two target DNAs showing a linear response over three logarithmic units (R^2^ = 0.9995 and 0.9965 for targets 1 and 2, respectively) were selected at a limit of detection of 20.46 pM and 19.09 pM (targets 1 and target 2, respectively). The limit of detection (LoD) is defined as the lowest concentration that can be determined from the control case (signal-to-noise ratio of 3). The similar dose responses and LoD of the two targets are due to the fact that the GC percentage of these targets is designed identically (Table S1, Supplementary Materials). Due to the enhanced assay capability of three-dimensional mesh-like scaffolds of hydrogels, streptavidin-conjugated encoded hydrogel microparticles resulted in a five-fold increase in sensitivity compared to microparticles with solid surfaces [45,46]. The specificity of the assay was assessed by measuring the fluorescent intensity of hydrogel microparticles for a total of four cases depending on the presence or the absence of the target at a 50 nM concentration (Figure S5, Supplementary Materials). The results showed that no false positive or negative signals were observed demonstrating the high specificity of the streptavidin-conjugated encoded hydrogel microparticles. 

We conducted an immunoassay based on the same particles used in DNA assays to demonstrate the flexibility of streptavidin-conjugated encoded hydrogel microparticles (Figure 3c). Ovalbumin, well known as an allergy-causing protein, was selected as a target [47]. Similar to the biotinylated DNA probe attachment process, the biotinylated antibody reacted with the particles within an hour, demonstrating that the use of streptavidin-conjugated encoded hydrogel microparticles in biomolecule assays is an effective method for rapid probe-set modification. The sensitivity and dynamic range were assessed by using Alexa 488-labeled ovalbumin at concentrations ranging from 240 to 62,500 pg/mL. Ovalbumin showed a linear response over 2.5 logarithmic units (R^2^ = 0.9992). Notably, the LoD of ovalbumin using streptavidin-conjugated encoded hydrogel microparticles was determined as 28.51 pg/mL, which is a 10-fold decrease compared to that of the magnetic bead-based assay [48]. 

### 3.4. Multiplex Detection of Cancer-Related miRNAs

To assess the feasibility of streptavidin-conjugated encoded hydrogel microparticles for clinical use, we selected two cancer-related miRNAs, hsa-miR-451a and hsa-miR-6880-5p [49,50]. These miRNAs are known to be dysregulated in pancreatic cancer, which is one of the most fatal human cancers and causes 227,000 deaths per year worldwide [51]. Different from the proof-of-concept studies that used fluorescently-labeled targets, an additional labeling method is required for the clinical assays to identify the target binding events inside the particle because targets are untreated with other labeling substances remaining in their original form in body fluids. 

**Figure 4:**
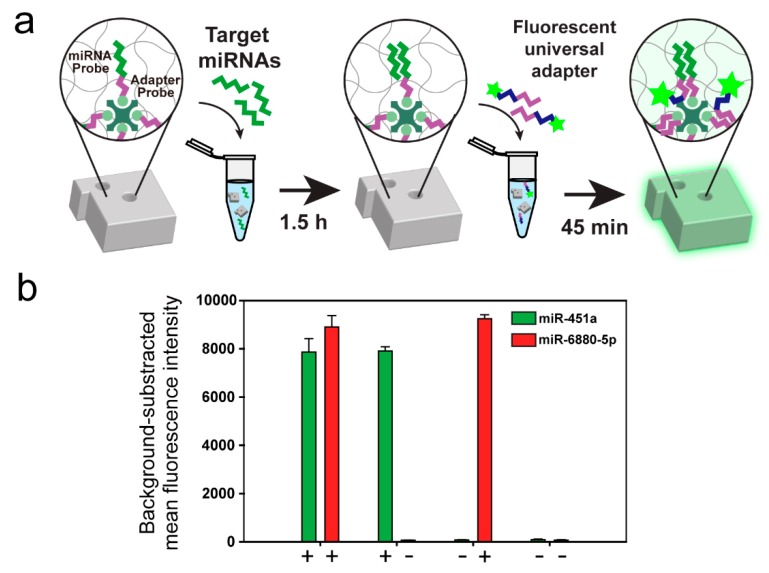
Multiplex detection of cancer-related miRNAs using streptavidin-conjugated encoded hydrogel microparticles. (**a**) Schematic illustration of the miRNA assay protocol. (**b**) Specificity test using two cancer-related miRNAs. The plus and minus signs represent the presence (+) or absence (−) of the target.

Selecting an appropriate labeling method is important as it mainly affects the assay performance including sensitivity, specificity, and dynamic range [52,53]. Among the suggested labeling methods, fluorescent labeling has been extensively used because it provides a sensitive and robust quantification of target binding events [14,17,54]. Therefore, we adopted a previously established fluorescent labeling procedure for the miRNA assay (Figure 4a) [55]. Briefly, the biotinylated capture probe consists of two parts: (1) a miRNA binding sequence for capturing target miRNA, and (2) a universal adapter binding sequence for fluorescent reporting. After hybridizing with target miRNA and particles, the fluorescently-labeled universal adapter was introduced into the mixture to form a linkage with the hybridized target miRNA by enzymatic ligation. Due to the low melting temperature of the fluorescently-labeled universal adapter, it cannot remain hybridized in the absence of the target. Therefore, the fluorescence intensity determined from the universal adapter in the hydrogel microparticles is proportional to the number of hybridized target miRNAs. For the detection of the two cancer-related miRNA biomarkers, we independently spiked each miRNA at concentrations ranging from 0.08 nM to 50 nM (Figure S6, Supplementary Material). Two miRNAs showed linear responses as a function of the different concentrations with LoDs of 28.06 pM and 33.49 pM for miR-451a and mir-6880-5p, respectively. A decreased sensitivity compared to that of the DNA assay from a previous study that used different assay conditions was observed as we adopted highly-stringent hybridization conditions in the miRNA assay to clearly discriminate the targets with similar sequences as a highly-specific detection is required in the clinical settings. To assess specificity, we performed multiplex detection in a total of fourcases depending on the presence or absence of two cancer-related miRNAs at a concentration of 5 nM (Figure 4b). These results confirm that the streptavidin-conjugated encoded hydrogel microparticles are able to quantify cancer-related miRNAs without cross-reactivity. Sensitivity could be further increased using streptavidin–phycoerythrin as a fluorophore (SA–PE) as it possesses a 30 times higher fluorescent yield than that of fluorescein, such as the FAM dye used in this study [56].

## 4. Conclusions

Using the unreacted double bonds remaining in the encoded hydrogel microparticles after the fabrication process, we directly conjugated streptavidin to the encoded hydrogel microparticles via the aza-Michael addition click reaction. By incorporating streptavidin into the encoded hydrogel microparticles, highly flexible multiplex bioassays were achieved with a high assay capability as the streptavidin–biotin reaction enables rapid probe-set modification and three-dimensional mesh-like scaffolds provide enhanced binding kinetics. Streptavidin-conjugated encoded hydrogel microparticles demonstrated high flexibility and sensitivity in assessing DNA and protein, and were successfully used in the multiplex detection of cancer-related miRNAs. We believe that the streptavidin-conjugated encoded hydrogel microparticle-based bioassays can become a feasible platform for disease-related biomarker quantification where rapid probe-set modifications are required to achieve a high sensitivity and specificity of diagnosis. Future studies will be aimed at demonstrating the reliability of the streptavidin-conjugated encoded hydrogel microparticle-based assays in plasma or serum, and to improve sensitivity with respect to the detection of rare biomarkers in body fluids.

## Supplementary Materials

The following are available online at https://www.mdpi.com/2073-4360/12/3/546/s1, Figure S1: Kinetics of the aza-Michael addition reaction, Figure S2: Fluorescence intensity of particles versus streptavidin concentration, Figure S3: Stability of streptavidin conjugated in encoded hydrogel microparticles, Figure S4: DNA probe density of particles as a function of streptavidin concentration, Figure S5: Specificity test using two DNA targets, Figure S6: Standard calibration curves of two cancer-related miRNAs, and Table S1: Sequences of DNA and miRNA targets, probes, and the universal adapter.

## References

[1] Nolan J.P., Sklar L.A. (2002). Suspension array technology: Evolution of the flat-array paradigm. Trends Biotechnol..

[2] Zhao Y., Zhao X., Hu J., Xu M., Zhao W., Sun L., Zhu C., Xu H., Gu Z. (2009). Encoded porous beads for label-free multiplex detection of tumor markers. Adv. Mater..

[3] Kim S.H., Shim J.W., Yang S.M. (2011). Microfluidic multicolor encoding of microspheres with nanoscopic surface complexity for multiplex immunoassays. Angew. Chem., Int. Ed..

[4] Zhao X.-W., Liu Z.-B., Yang H., Nagai K., Zhao Y.-H., Gu Z.-Z. (2006). Uniformly colorized beads for multiplex immunoassay. Chem. Mater..

[5] Jun B.-H., Kim J.-H., Park H., Kim J.-S., Yu K.-N., Lee S.-M., Choi H., Kwak S.-Y., Kim Y.-K., Jeong D.H. (2007). Surface-enhanced Raman spectroscopic-encoded beads for multiplex immunoassay. J. Comb. Chem..

[6] Elshal M.F., McCoy J.P. (2006). Multiplex bead array assays: Performance evaluation and comparison of sensitivity to ELISA. Methods.

[7] Dunbar S.A. (2006). Applications of Luminex® xMAP™ technology for rapid, high-throughput multiplexed nucleic acid detection. Clin. Chim. Acta.

[8] Pregibon D.C., Doyle P.S. (2009). Optimization of encoded hydrogel particles for nucleic acid quantification. Anal. Chem..

[9] Lee W., Choi D., Kim J.-H., Koh W.-G. (2008). Suspension arrays of hydrogel microparticles prepared by photopatterning for multiplexed protein-based bioassays. Biomed. Microdevices.

[10] Roh Y.H., Lee H.J., Bong K.W. (2019). Microfluidic Fabrication of Encoded Hydrogel Microparticles for Application in Multiplex Immunoassay. BioChip J..

[11] Kingsmore S.F. (2006). Multiplexed protein measurement: Technologies and applications of protein and antibody arrays. Nat. Rev. Drug Discov..

[12] Meiring J.E., Schmid M.J., Grayson S.M., Rathsack B.M., Johnson D.M., Kirby R., Kannappan R., Manthiram K., Hsia B., Hogan Z.L. (2004). Hydrogel biosensor array platform indexed by shape. Chem. Mater..

[13] Park S., Lee H.J., Koh W.-G. (2012). Multiplex immunoassay platforms based on shape-coded poly (ethylene glycol) hydrogel microparticles incorporating acrylic acid. Sensors.

[14] Appleyard D.C., Chapin S.C., Srinivas R.L., Doyle P.S. (2011). Bar-coded hydrogel microparticles for protein detection: Synthesis, assay and scanning. Nat. Protoc..

[15] Dendukuri D., Gu S.S., Pregibon D.C., Hatton T.A., Doyle P.S. (2007). Stop-flow lithography in a microfluidic device. Lab Chip.

[16] Roh Y.H., Sim S.J., Cho I.-J., Choi N., Bong K.W. (2016). Vertically encoded tetragonal hydrogel microparticles for multiplexed detection of miRNAs associated with Alzheimer’s disease. Analyst.

[17] Lee H.J., Roh Y.H., Kim H.U., Kim S.M., Bong K.W. (2019). Multiplexed immunoassay using post-synthesis functionalized hydrogel microparticles. Lab Chip.

[18] Roh Y.H., Lee H.J., Moon H.J., Kim S.M., Bong K.W. (2019). Post-synthesis functionalized hydrogel microparticles for high performance microRNA detection. Anal. Chim. Acta.

[19] Sharma P., Sahni N.S., Tibshirani R., Skaane P., Urdal P., Berghagen H., Jensen M., Kristiansen L., Moen C., Sharma P. (2005). Early detection of breast cancer based on gene-expression patterns in peripheral blood cells. Breast Cancer Res..

[20] Fung K.Y., Tabor B., Buckley M.J., Priebe I.K., Purins L., Pompeia C., Brierley G.V., Lockett T., Gibbs P., Tie J. (2015). Blood-based protein biomarker panel for the detection of colorectal cancer. PLoS ONE.

[21] Srisa-Art M., Dyson E.C., deMello A.J., Edel J.B. (2008). Monitoring of real-time streptavidin− biotin binding kinetics using droplet microfluidics. Anal. Chem..

[22] González M.n., Argaraña C.E., Fidelio G.D. (1999). Extremely high thermal stability of streptavidin and avidin upon biotin binding. Biomol. Eng..

[23] Ellison D., Beynon R.J., Hinton J., Hubbard S.J. (1995). Limited proteolysis of native proteins: The interaction between avidin and proteinase K. Protein Sci..

[24] Elia G. (2008). Biotinylation reagents for the study of cell surface proteins. Proteomics.

[25] Bong K.W., Kim J.J., Cho H., Lim E., Doyle P.S., Irimia D. (2015). Synthesis of Cell-Adhesive Anisotropic Multifunctional Particles by Stop Flow Lithography and Streptavidin–Biotin Interactions. Langmuir.

[26] Elliott J.E., Macdonald M., Nie J., Bowman C.N.J.P. (2004). Structure and swelling of poly (acrylic acid) hydrogels: Effect of pH, ionic strength, and dilution on the crosslinked polymer structure. Polymers.

[27] Graves H.C. (1988). The effect of surface charge on non-specific binding of rabbit immunoglobulin G in solid-phase immunoassays. J. Immunol. Methods.

[28] Cuatrecasas P., Parikh I. (1972). Adsorbents for affinity chromatography. Use of N-hydroxysuccinimide esters of agarose. Biochemistry.

[29] Dendukuri D., Pregibon D.C., Collins J., Hatton T.A., Doyle P.S. (2006). Continuous-flow lithography for high-throughput microparticle synthesis. Nat. Mater..

[30] Argarana C.E., Kuntz I.D., Birken S., Axel R., Cantor C.R. (1986). Molecular cloning and nucleotide sequence of the streptavidin gene. Nucleic Acids Res..

[31] Bosica G., Abdilla R. (2016). Aza-Michael mono-addition using acidic alumina under solventless conditions. Molecules.

[32] Konuray O., Fernández-Francos X., Ramis X., Serra À. (2018). State of the art in dual-curing acrylate systems. Polymers.

[33] Noordzij G., Wilsens C. (2019). Cascade aza-Michael addition-cyclizations; towards renewable and multifunctional carboxylic acids for melt-polycondensation. Front. Chem..

[34] Hoffmann C., Stuparu M.C., Daugaard A., Khan A. (2015). Aza-Michael addition reaction: Post-polymerization modification and preparation of PEI/PEG-based polyester hydrogels from enzymatically synthesized reactive polymers. J. Polym. Sci. Part A Polym. Chem..

[35] Genest A., Binauld S., Pouget E., Ganachaud F., Fleury E., Portinha D. (2017). Going beyond the barriers of aza-Michael reactions: Controlling the selectivity of acrylates towards primary amino-PDMS. Polym. Chem..

[36] Chen H., Huang R., Li Z., Zhu W., Chen J., Zhan Y., Jiang B. (2017). Selective lysine modification of native peptides via aza-Michael addition. Org. Biomol. Chem..

[37] Furman J.L., Kang M., Choi S., Cao Y., Wold E.D., Sun S.B., Smider V.V., Schultz P.G., Kim C.H. (2014). A genetically encoded aza-Michael acceptor for covalent cross-linking of protein–receptor complexes. J. Am. Chem. Soc..

[38] Almonte L., Lopez-Elvira E., Baró A.M. (2014). Surface-Charge Differentiation of Streptavidin and Avidin by Atomic Force Microscopy–Force Spectroscopy. ChemPhysChem.

[39] Echeverri M., Hamad C., Kyu T. (2014). Highly conductive, completely amorphous polymer electrolyte membranes fabricated through photo-polymerization of poly (ethylene glycol diacrylate) in mixtures of solid plasticizer and lithium salt. Solid State Ion..

[40] Krüger A., Bürkle A., Mangerich A., Hauser K. (2018). A combined approach of surface passivation and specific immobilization to study biomolecules by ATR-FTIR spectroscopy. Biomed. Spectrosc. Imaging.

[41] Tuschel D. (2014). Practical group theory and Raman spectroscopy, part II: Application of polarization. Spectroscopy.

[42] Kondo T., Hashimoto R., Ohrui Y., Sekioka R., Nogami T., Muta F., Seto Y. (2018). Analysis of chemical warfare agents by portable Raman spectrometer with both 785 nm and 1064 nm excitation. Forensic Sci. Int..

[43] Sorokin N., Chechetkin V., Pan’kov S., Somova O., Livshits M., Donnikov M., Turygin A., Barsky V., Zasedatelev A. (2006). Kinetics of hybridization on surface oligonucleotide microchips: Theory, experiment, and comparison with hybridization on gel-based microchips. J. Biomol. Struct. Dyn..

[44] Tavakoli J., Tang Y. (2017). Hydrogel based sensors for biomedical applications: An updated review. Polymers.

[45] Broder G.R., Ranasinghe R.T., She J.K., Banu S., Birtwell S.W., Cavalli G., Galitonov G.S., Holmes D., Martins H.F., MacDonald K.F. (2008). Diffractive micro bar codes for encoding of biomolecules in multiplexed assays. Anal. Chem..

[46] Zhi Z.-l., Morita Y., Yamamura S., Tamiya E. (2005). Microfabrication of encoded microparticle array for multiplexed DNA hybridization detection. Chem. Commun..

[47] Jung K.-H., Baek H., Shin D., Lee G., Park S., Lee S., Choi D., Kim W., Bae H. (2016). Protective effects of intratracheally-administered bee venom phospholipase A2 on ovalbumin-induced allergic asthma in mice. Toxins.

[48] Feng X.-L., Ren H.-L., Li Y.-S., Hu P., Zhou Y., Liu Z.-S., Yan D.-M., Hui Q., Liu D., Lin C. (2014). A magnetic particles-based chemiluminescence enzyme immunoassay for rapid detection of ovalbumin. Anal. Biochem..

[49] Thomas M.L., Marcato P. (2018). Epigenetic modifications as biomarkers of tumor development, therapy response, and recurrence across the cancer care continuum. Cancers.

[50] Guo R., Gu J., Zhang Z., Wang Y., Gu C. (2017). MiR-451 promotes cell proliferation and metastasis in pancreatic cancer through targeting CAB39. BioMed Res. Int..

[51] Raimondi S., Maisonneuve P., Lowenfels A.B. (2009). Epidemiology of pancreatic cancer: An overview. Nat. Rev. Gastroenterol. Hepatol..

[52] Zhou L., Ding F., Chen H., Ding W., Zhang W., Chou S.Y. (2012). Enhancement of immunoassay’s fluorescence and detection sensitivity using three-dimensional plasmonic nano-antenna-dots array. Anal. Chem..

[53] Cho D.G., Yoo H., Lee H., Choi Y.K., Lee M., Ahn D.J., Hong S. (2018). High-Speed Lateral Flow Strategy for a Fast Biosensing with an Improved Selectivity and Binding Affinity. Sensors.

[54] Huang J., Park J.H., Back S.H., Feng Y., Cui C., Jin L.Y., Ahn D.J. (2018). Mercury ion–DNA specificity triggers a distinctive photoluminescence depression in organic semiconductor probes guided with a thymine-rich oligonucleotide sequence. Nanoscale.

[55] Chapin S.C., Appleyard D.C., Pregibon D.C., Doyle P.S. (2011). Rapid microRNA profiling on encoded gel microparticles. Angew. Chem. Int. Ed..

[56] Hermanson G.T. (2013). Bioconjugate Techniques.

