# Supplementary material for: Direct Conjugation of Streptavidin to Encoded Hydrogel Microparticles for Multiplex Biomolecule Detection with Rapid Probe-Set Modification
Source: Polymers (Basel). 2020 Mar 3;12(3):546. doi: 10.3390/polym12030546 (PMC7182943; doi:10.3390/polym12030546)
Supplement: Supplementary file 1 [file polymers-12-00546-s001.zip › polymers-692473-SI/polymers-692473-supplementary.docx]

Supplementary Information for

**Direct Conjugation of Streptavidin to Encoded Hydrogel Microparticles for Multiplex Biomolecule Detection with Rapid Probe-set Modification**

Yoon Ho Roh^1^, Ju Yeon Kim^1^, Seok Joon Mun^1^, Hye Sun Lee^2^, Changhyun Hwang^1^, Kyong Hwa Park^2^ and Ki Wan Bong^1,^*

1. Department of Chemical and Biological Engineering, Korea University, 145, Anam-ro, Seongbuk-gu, Seoul, 02841, Republic of Korea
2. Division of Oncology/Hematology, Department of Internal Medicine, Korea University College of Medicine, 145, Anam-ro, Seongbuk-gu, Seoul, 02841, Republic of Korea

**
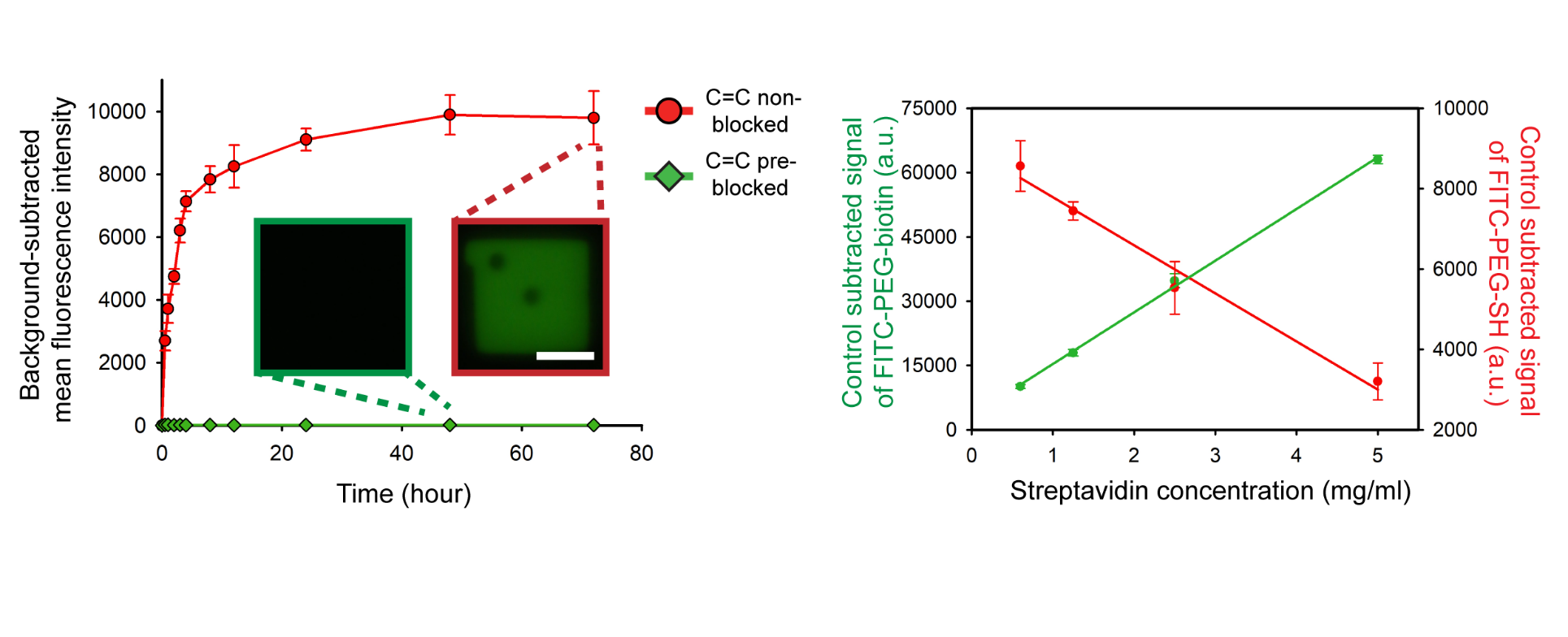
**

**Figure S1.** Kinetics of aza-Michael addition reaction. Red line represents the kinetics of reaction in the particles without post-processing and green line represents the kinetics of reaction in the particles where the unreacted double bonds are blocked in advance by using SH-PEG. Scale bar is 25 μm.


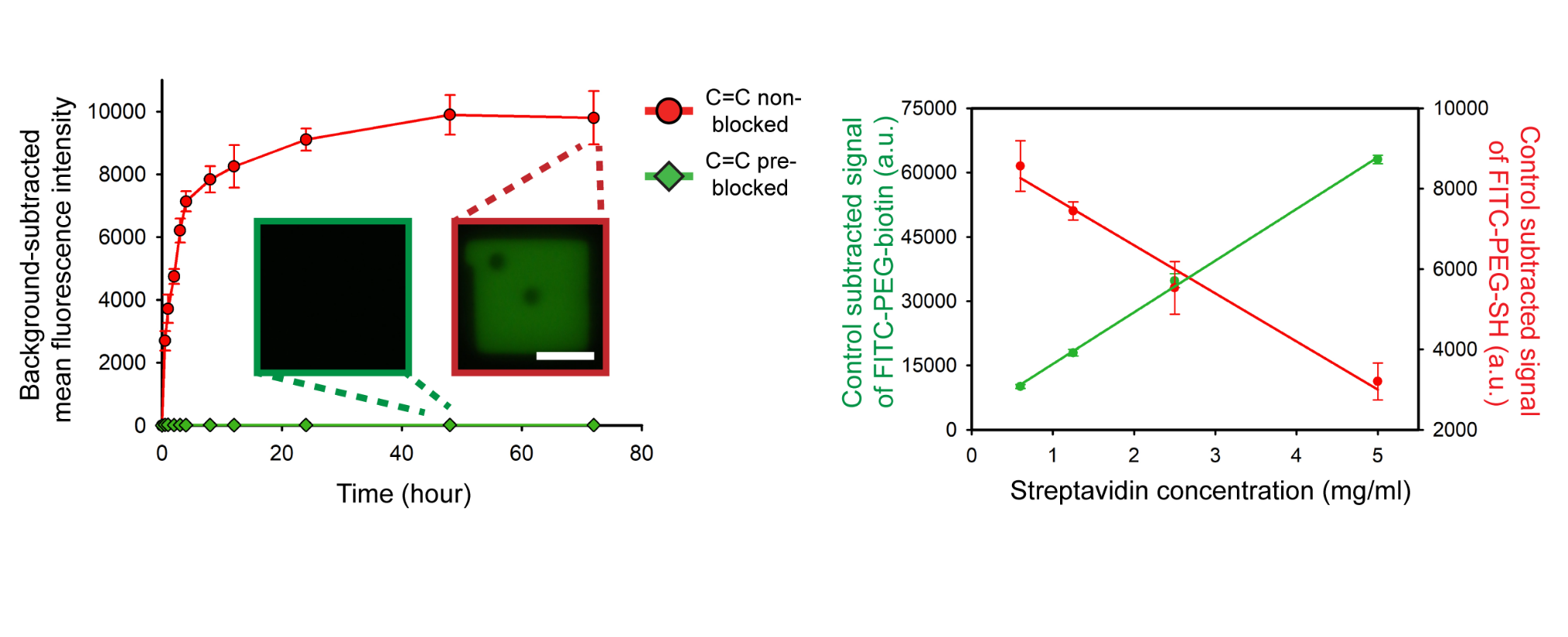


**Figure S2.** Fluorescence intensity of particles according to the streptavidin concentration. Green line represents the fluorescence intensity that represents the streptavidin concentration and red line represents the fluorescence intensity that represents the remaining unreacted double bonds after the reaction with streptavidin.


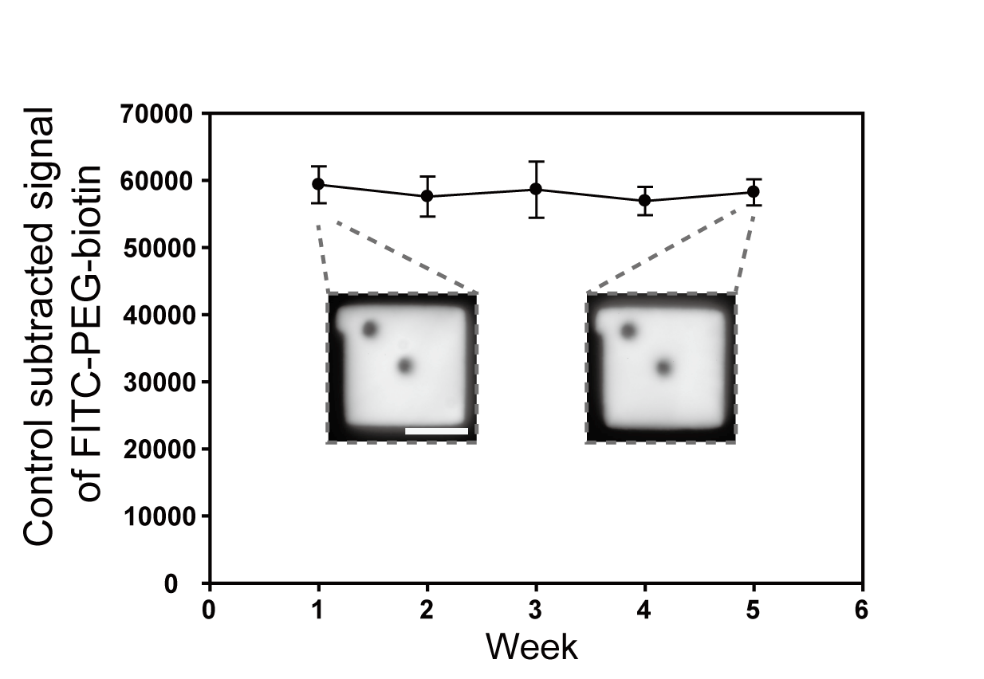


**Figure S3.** Stability of streptavidin conjugated in encoded hydrogel microparticles. Streptavidin was stably conjugated in particles for at least 5 weeks. Scale bar is 25 μm.


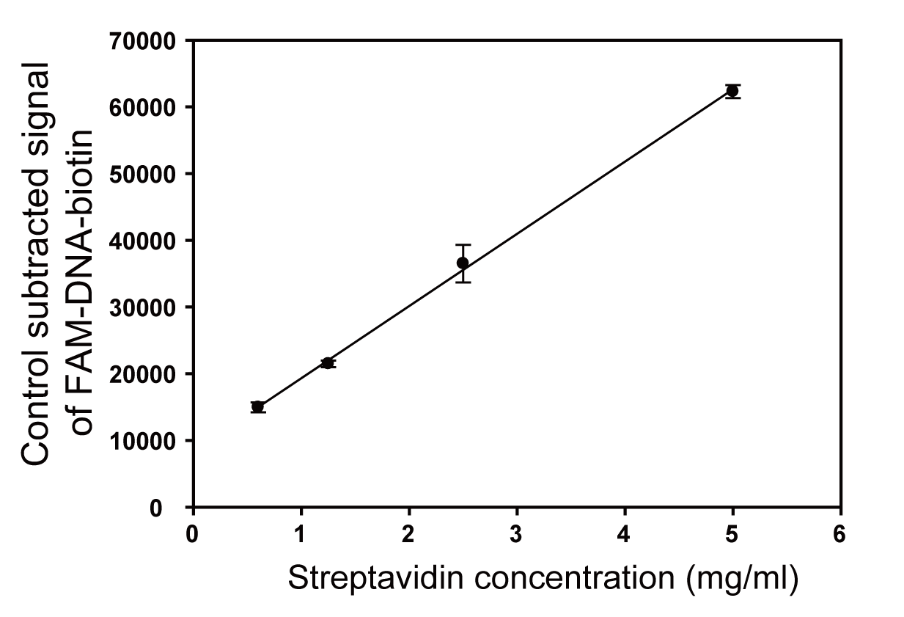


**Figure S4.** DNA probe density of particles according to the streptavidin concentration. DNA attached with 6-FAM fluorescent dye was incorporated into the encoded hydrogel microparticles which were conjugated with various concentration of streptavidin. Fluorescent intensity of FAM attached DNA probe increased linearly according to the streptavidin concentration.


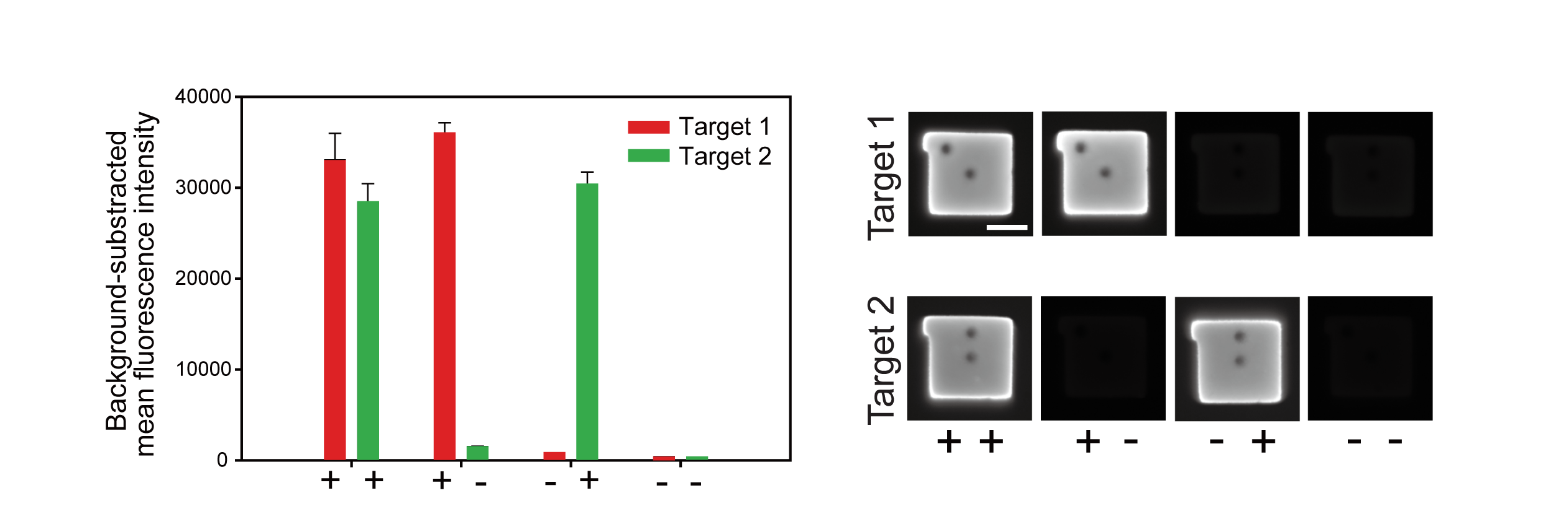
**Figure S5.** Specificity test using two DNA targets. Bar graph showing background-subtracted mean fluorescence intensity of 4 cases depending on the presence or absence of two DNA targets (left) and fluorescence micrographs of streptavidin-conjugated hydrogel microparticles (right). The plus and minus sign represent the presence (+) and absence (-) of the target. Scale bar is 25 μm.


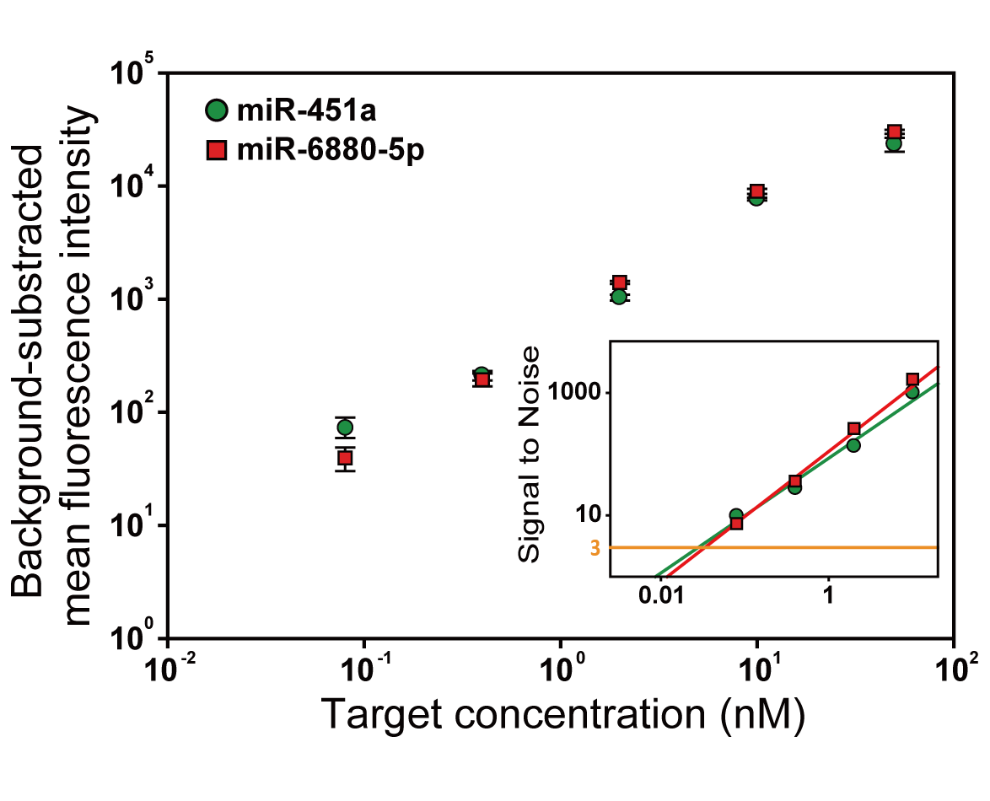


**Figure S6.** Standard calibration curves of two cancer-related miRNAs.

**Estimation of the amount of double bonds remaining inside the hydrogel particles and molar ratio of acrylate to amine in reaction**

To estimate the amount of double bonds in encoded hydrogel microparticles, the reaction volume to be polymerized into a single particle was first defined as the dimension of hydrogel particles (50 × 50 × 20 μm). This is because polymerization occurs in a confined area due to the shape-defined UV according to the shape of photomask. Since the PEGDA 700 is mostly related to the formation of hydrogel networks and remnant double bonds after polymerization, we only considered the portion of PEGDA 700 among the substances in the monomer. By multiplying the reaction volume by the portion of PEGDA 700 (20%), we obtained 10 pL. Then, the volume of PEGDA 700, which participates in reaction to be polymerized into a single particle, was converted to the number of moles by using the density and molecular weight of PEGDA 700. The conversion rate of polymerization is about 20%, which was previously determined under the similar polymerization condition of this work [1]. Therefore, estimated amount of double bonds remaining inside the hydrogel particles was ~12.8 pmol, which was obtained by multiplying 0.8 times by the number of moles of PEGDA 700 in reaction volume.

To estimate the molar ratio of acrylate to amine during the aza-Michael addition reaction, we first calculated the amount of primary amine in streptavidin that participates in the reaction. Mole number of streptavidin was obtained by dividing amount of streptavidin used in the reaction (500 μg) by a molecular weight. Since the streptavidin has four primary amines, total amount of amine was calculated as 36 nmol. The amount of acrylates that participates in the reaction was 75.6 nmol which was calculated by multiplying total numbers of particles in reaction (6000 particles) by the amount of acrylate group remaining in a single particle. Therefore, the molar ratio of acrylate to amine is ~ 2:1.

**Table S1.** Sequences of DNA and miRNA targets, probes and universal adapter

| **Name** | | **Sequence** |
| --- | --- | --- |
| Target 1 | Target | 5’-FAM-TCA CCA ATC GCA CAC TAC TCA -3’ |
|  | Probe | /5Biosg/ TTT TTT TTT TGA GTA GTG TGC GAT TGG TGA |
| Target 2 | Target | 5’-FAM-CCC AAG AAC ACG ATA CCA GAA -3’ |
|  | Probe | /5Biosg/ TTT TTT TTT TTC TGG TAT CGT GTT CTT GGG |
| miR-451a | Target | 5’- AAA CCG UUA CCA UUA CUG AGU U -3’ |
|  | Probe | /5ThioMC6-D/ G**AT ATA TTT TA**A ACT CAG TAA TGG TAA CGG TTT |
| miR-6880-5p | Target | 5’- UGG UGG AGG AAG AGG GCA GCU C -3’ |
|  | Probe | /5ThioMC6-D/ G**AT ATA TTT TA**G AGC TGC CCT CTT CCT CCA CCA |
| Universal adapter | | /5Phos/**TAAAATATAT**AAAAAAAAAAAA/3FAM/ |

Red color-marked bases represent a sequence of the universal adapter.

**References**

[1] Dendukuri, D.; Pregibon, D. C.; Collins, J.; Hatton, T. A.; Doyle, P. S. Continuous-flow lithography for high-throughput microparticle synthesis. *Nat. Mater.* **2006**, *5*, 365-369.
